# RE: A case of treatment-resistant depression in an older adult and a discussion of treatment options

**DOI:** 10.1192/bjb.2023.21

**Published:** 2023-06

**Authors:** Richard Braithwaite

**Affiliations:** Consultant Psychiatrist, Sussex Partnership NHS Foundation Trust, UK. Email: richard.braithwaite@nhs.net

## Mirtazapine augmentation of other antidepressants is ineffective

Pope and colleagues' discussion of the treatment options in refractory depressive disorder^1^ uses a fictional case history to promote the augmentation of unsuccessful venlafaxine monotherapy with the presynaptic α2 autoreceptor antagonist mirtazapine. In support, the authors cite a 2016 systematic review by Henssler and colleagues,^2^ which they state concluded that combining mirtazapine with selective serotonin reuptake inhibitors (SSRIs) or serotonin and noradrenaline reuptake inhibitors (SNRIs) is more effective than other antidepressant combinations. I fear they have misinterpreted that review's findings, failed to take account of the difference between augmentation and combination treatments, and overlooked recent, more robust, evidence.

Data from ten randomised controlled trials (RCTs) that combined α2 antagonist (A2A) drugs with re-uptake inhibitors were pooled in the meta-analysis by Henssler et al, which suggested that combinations involving an A2A were effective, contrasting with a similar analysis in the paper showing that combinations not involving an A2A were ineffective.^2^ Only three RCTs involved mirtazapine; the others used trazodone or mianserin instead.^2^ This group of drugs is heterogeneous, with each agent acting to varying degrees across a vast array of neuroreceptors and the only common feature being antagonism of the α2 receptor, an action that may or may not be related to therapeutic effect. The reviewers' decision to pool data according to this grouping is therefore questionable, but, crucially, Pope and colleagues have misquoted the review's stated conclusions, which refer to the A2A group as a whole and not to mirtazapine specifically.

A distinction must be drawn between augmentation (addition of a second intervention to a treatment that has been ineffective on its own) and combination (simultaneous, or near simultaneous, initiation of two treatments, neither of which is known to have already been ineffective) strategies. The fictional patient described by Pope et al has venlafaxine augmented with mirtazapine, yet the cited systematic review upon which they base this strategy^2^ found only one RCT comparing mirtazapine with placebo augmentation of another agent. That US study was very small and funded by Organon, the original manufacturer of mirtazapine. Seven of the 11 patients given mirtazapine augmentation responded at 4 weeks, after previously failing to improve on the initial antidepressant alone, compared with three of 15 patients on placebo augmentation.^3^ Remarkably, this finding became the sole basis upon which the UK National Institute for Clinical Excellence (NICE) recommended adding (rather than switching to) mirtazapine following an unsuccessful trial of an SSRI or SNRI in the 2004 edition of its guideline for treating depressive disorder.^4^ This led to widespread adoption of this weakly evidenced practice in the UK and elsewhere.

In a more recent and much larger multicentre double-blind RCT that sampled 431 patients who had not responded to SSRI or SNRI monotherapy, UK researchers found a statistically significant difference in mean Beck Depression Inventory scores between the mirtazapine and placebo augmentation groups at 12 weeks.^5^ However, the observed difference of 1.7 points did not come close to the pre-defined cut-off for a clinically significant effect. Furthermore, there were no statistically significant differences in response or remission rates between the groups, such that the authors concluded that adding mirtazapine to another antidepressant that has already proved unsuccessful cannot be recommended.^5^ Despite this, NICE continues to advise mirtazapine augmentation in its 2022 guideline.^6^

The evidence for combining mirtazapine with another, previously untried, antidepressant is marginally better and is largely derived from two small Canadian RCTs.^7,8^ Both were manufacturer-funded and studied mirtazapine or placebo combined with paroxetine^7^ or fluoxetine.^8^ It is probable most subjects were naïve to both drugs at the beginning of each study, with fluoxetine non-responders specifically excluded from the latter.^8^ The statistically significant findings in terms of response (but not remission) rates^7,8^ suggest that this combination may be slightly more effective than SSRI monotherapy, but crucially this conclusion does not extend to patients who have already failed to respond to the SSRI on its own. A follow-on observational phase, using mirtazapine augmentation in paroxetine monotherapy non-responders, had no control arm,^7^ so is of little import. Another two blind RCTs,^9,10^ each of which compared the venlafaxine–mirtazapine combination against monotherapy with a drug from a different class entirely, are difficult to interpret in practice, whereas an open-label study^11^ lacked any placebo control.

Polypharmacy is potentially dangerous. As Pope et al themselves note, taking these drugs together can lead to serotonin syndrome, as well as an increased range of other serious side-effects.^1^ Unsurprisingly, the aforementioned multicentre study found a significantly higher rate of adverse effects in the active augmentation group compared with monotherapy plus placebo.^5^

Psychiatrists need to change their practice, to protect patients from the dual harms of ineffective treatment and enhanced side-effects. To this end, NICE guidance needs to be updated, with removal of the advice to augment an unsuccessful SSRI or SNRI with mirtazapine. Rather, it could suggest, with reservations concerning the relatively weak evidence base, that switching to a fresh combination involving mirtazapine might be considered. I fear that, by publishing a fictional case in which the patient responds to addition of mirtazapine,^1^ Pope and colleagues have perpetuated the widely believed myth that this augmentation strategy is effective.
